# Effects of neurofeedback training combined with transcranial direct current stimulation on motor imagery: A randomized controlled trial

**DOI:** 10.3389/fnins.2023.1148336

**Published:** 2023-03-02

**Authors:** Shun Sawai, Shin Murata, Shoya Fujikawa, Ryosuke Yamamoto, Keisuke Shima, Hideki Nakano

**Affiliations:** ^1^Graduate School of Health Sciences, Kyoto Tachibana University, Kyoto, Japan; ^2^Department of Rehabilitation, Kyoto Kuno Hospital, Kyoto, Japan; ^3^Department of Physical Therapy, Faculty of Health Sciences, Kyoto Tachibana University, Kyoto, Japan; ^4^Department of Rehabilitation, Tesseikai Neurosurgical Hospital, Shijonawate, Japan; ^5^Graduate School of Environment and Information Sciences, Yokohama National University, Yokohama, Japan

**Keywords:** motor imagery, neurofeedback, brain-computer interface, tDCS, combination

## Abstract

**Introduction:**

Neurofeedback (NFB) training and transcranial direct current stimulation (tDCS) have been shown to individually improve motor imagery (MI) abilities. However, the effect of combining both of them with MI has not been verified. Therefore, the aim of this study was to examine the effect of applying tDCS directly before MI with NFB.

**Methods:**

Participants were divided into an NFB group (*n* = 10) that performed MI with NFB and an NFB + tDCS group (*n* = 10) that received tDCS for 10 min before MI with NFB. Both groups performed 60 MI trials with NFB. The MI task was performed 20 times without NFB before and after training, and μ-event-related desynchronization (ERD) and vividness MI were evaluated.

**Results:**

μ-ERD increased significantly in the NFB + tDCS group compared to the NFB group. MI vividness significantly increased before and after training.

**Discussion:**

Transcranial direct current stimulation and NFB modulate different processes with respect to MI ability improvement; hence, their combination might further improve MI performance. The results of this study indicate that the combination of NFB and tDCS for MI is more effective in improving MI abilities than applying them individually.

## 1. Introduction

Motor imagery (MI) is the cognitive process of imagining the execution of a movement without actually moving (Lotze and Halsband, 2006; Olsson and Nyberg, 2010); it can improve skill (Ladda et al., 2021) and muscle strength (Yue and Cole, 1992) despite the lack of movement. Therefore, it is used in rehabilitation and sports training (MacIntyre et al., 2018). It has been shown that MI shares some of the neural activities with actual motor execution (Lotze et al., 1999; Hanakawa et al., 2003; Nakano et al., 2012), which likely supports the effectiveness of MI. Particularly, MI involves abundant neural activity (Ruffino et al., 2016); therefore, it promotes neuroplasticity and is used in the rehabilitation of neurological diseases such as stroke (Guerra et al., 2017) and Parkinson’s disease (Tinaz et al., 2022). Further, it improves upper limb function in patients who have had stroke (Villa-Berges et al., 2023) and reduces bradykinesia in patients with Parkinson’s disease (Tamir et al., 2007). Specifically, upper limb function is less likely to improve compared with lower limb function in patients who have had stroke (Liu et al., 2012), and MI may be an effective novel intervention method. However, as MI is a purely mental process, it does not provide any feedback (Bai et al., 2014); thus, its effectiveness varies among individuals (van der Meulen et al., 2014). This issue can be overcome by using neurofeedback (NFB) (Hwang et al., 2009; Yu et al., 2015; Nakano et al., 2018). NFB is a technique for improving performance by feeding back brain activity recorded during task execution to the participants (Hampson et al., 2020). In particular, closed-loop adaptations of NFB are used for MI; they promote the active regulation of brain activity (Al-Wasity et al., 2021; Grigorev et al., 2021) by providing brain activity (Sitaram et al., 2019) and performance feedback (Riahi et al., 2022). Thus, positive effects of NFB on MI have been reported. Furthermore, NFB improves MI in stroke (Dettmers et al., 2016) and Parkinson’s disease (Tinaz et al., 2018, 2022), and may also address the problem of MI in patients with neurological diseases. Neurological diseases, such as stroke, decrease MI ability (Dettmers et al., 2012; Braun et al., 2017); thus, NFB for MI may be an effective novel treatment method.

On the other hand, transcranial direct current stimulation (tDCS), a method to directly modify brain states, has attracted increasing attention. tDCS is a reversible, focal, non-invasive brain stimulation technique that modulates neuroplasticity by applying a direct current to the head *via* electrodes (Nitsche and Paulus, 2001). In neurons, it changes the resting membrane potential toward depolarization or hyperpolarization, depending on the direction of the current flow relative to the direction of the axons (Lefaucheur et al., 2017). Further, it has been shown that anodal tDCS increases cortical excitability immediately below the electrode, whereas cathodal tDCS decreases cortical excitability (Nitsche and Paulus, 2000). These neural effects affect exercise performance, motor learning (Reis and Fritsch, 2011; Buch et al., 2017), and cognitive function (Pope et al., 2015; Imburgio and Orr, 2018). In addition, tDCS alters various symptoms, including attention function in children with paralysis (Alharbi et al., 2021), mental status in patients with depression (Palm et al., 2016), and pain intensity in patients with chronic pain (Pacheco-Barrios et al., 2020). tDCS has also been used to treat neurological diseases because it modulates neuroplasticity (Di Pino et al., 2014). By directly modulating brain states, it improves motor functions, such as gait (Chieffo et al., 2016) and upper limb function (Pires et al., 2023).

Recently, the effects of the combination of MI and tDCS have been investigated. In healthy young adults, the combination of MI and tDCS enhances event-related desynchronization (ERD) in the primary motor cortex (M1) (Wei et al., 2013; Xie et al., 2021) and improves connectivity in the sensorimotor cortex (Baxter et al., 2017). These changes in brain states improve MI performance (Moghadas Tabrizi et al., 2019). In addition, in stroke patients, the combination of MI and tDCS promotes neuroplasticity (Hong et al., 2017) and improves the Fugl-Meyer Assessment score (Chew et al., 2020; Kashoo et al., 2022). While it has been reported that the combinations of MI and NFB as well as MI and tDCS improve MI performance, the effect of combining both NFB and tDCS with MI has not yet been verified, although this combination may provide a new approach to maximizing MI performance.

Therefore, this study aimed to investigate the combined effect of tDCS and NFB in MI tasks in healthy young adults.

## 2. Materials and methods

### 2.1. Participants

Twenty healthy young men (age: 20.20 ± 0.70 years, height: 172.65 ± 6.77 cm, and body weight: 63.00 ± 8.52 kg) without a history of motor or cognitive dysfunction and metal implants in the head were recruited for this study. Supplementary Table 1 summarizes their demographic data. The Edinburgh Handedness Inventory (Oldfield, 1971) was used to confirm that all participants were right-handed.

This study was conducted in accordance with the Helsinki Declaration, and informed consent was obtained from all participants. Additionally, this research was approved by the Research Ethics Committee of Kyoto Tachibana University (approval no. 21-47).

### 2.2. Sample size calculation

The sample size was determined as 16 using the G*Power software (Faul et al., 2007) by considering an effect size as “medium” at 0.40 (Cohen, 1998), α = 0.05, and power (1-β) = 0.80 at 95% confidence level.

### 2.3. Study protocol

**FIGURE 1:**
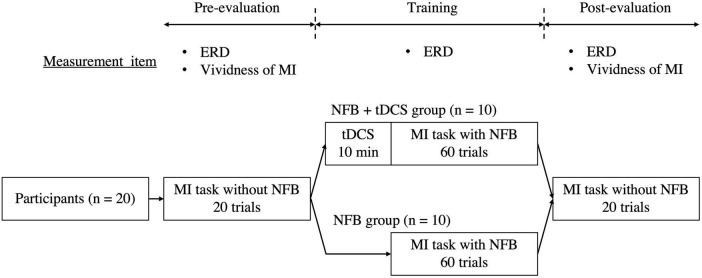
Study protocol. Participants were divided into an NFB group (*n* = 10) performing the MI task with NFB alone and a tDCS + NFB group (*n* = 10) performing the MI task with NFB after 10 min of tDCS. ERD and MI vividness were evaluated before and after the training phase. In the training phase, ERD during the MI task was also evaluated. MI, motor imagery; NFB, neurofeedback; tDCS, transcranial direct current stimulation; ERD, event-related desynchronization.

This study was a randomized controlled trial. First, in the pre-evaluation phase, all participants performed the MI task 20 times without NFB. Second, for the training phase, they were randomly divided into two groups as follows: (i) NFB group (*n* = 10, age: 20.40 ± 0.70 years, height: 173.70 ± 4.50 cm, and body weight: 64.40 ± 5.42 kg) that performed 60 MI tasks with NFB and (ii) tDCS + NFB group (*n* = 10, age: 20.00 ± 0.67 years, height: 171.60 ± 8.60 cm, and body weight: 61.60 ± 10.93 kg) that performed 60 MI tasks with NFB after receiving tDCS for 10 min (Yamaguchi et al., 2020). Finally, in the post-evaluation phase, all participants performed the MI task without NFB 20 times again. The self-reported MI vividness was assessed during the pre- and post-evaluation phases. Additionally, EEG was recorded during the MI tasks and ERD was evaluated during the pre-evaluation, training, and post-evaluation phases (Figure 1).

### 2.4. Motor imagery

**FIGURE 2:**
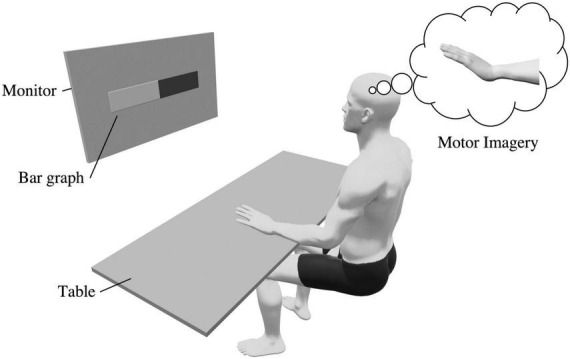
Experimental setup. Participants were seated in a chair, placed their left hand on a desk, and imagined a kinesthetic wrist dorsiflexion movement. During the training phase, a bar graph was displayed on the monitor and the ERD was fed back to the participant.

During the training phase, a bar graph was displayed on a monitor to provide real-time feedback based on the ERD (Figure 2). Participants were asked to imagine dorsiflexion of the left wrist joint at 100% maximum voluntary contraction strength from a first-person perspective. The imagery task consisted of a resting period (5 s) followed by an imagery period (5 s), and participants repeated the imagery task 20 times during the pre- and post-evaluation phases. In the training phase, three sets of 20 imagery tasks were performed. Several MI studies consisted of short repetitions of the imagery and rest periods (Xie et al., 2021; Sawai et al., 2022); we followed a similar protocol. The degree of MI improves with its repetition (Pineda et al., 2003); therefore, few trials may erase the effect of tDCS. Conversely, numerous trials can cause mental fatigue, which may reduce the degree of MI (Nakashima et al., 2021). Therefore, we set the number of trials at 60, which has been used in previous studies (Maghsoudi and Shalbaf, 2022; Sawai et al., 2022).

### 2.5. Neurofeedback

In the training phase, EEG was recorded during MI and ERD values were fed back to the participants in real-time using an electroencephalograph (EEG-9100; Nihon Kohden Corp., Tokyo, Japan) and an active dry electrodes system (Miyuki Giken, Co., Ltd., Tokyo, Japan). EEG was recorded from 19 channel locations (Fp1, Fp2, F7, F3, Fz, F4, F8, T3, C3, Cz, C4, T4, T5, P3, Pz, P4, T6, O1, and O2) according to the international 10–20 system, and reference electrodes were attached to both earlobes. The sampling rate was set at 1,000 Hz. The EEG data were analyzed using Microsoft Visual Studio (Microsoft Corp., Redmond, WA, USA), and the ERD in the μ-wave band (8–13 Hz) of the sensorimotor-related region (C4) was calculated using frequency analysis. ERD in the μ-wave band of C4 is associated with MI (Pineda et al., 2003; Enomae et al., 2017). The ERD *E(t)* at a given time *t* was calculated using the following equation using the resting μ-wave activity *R*_rest_ and the μ-wave activity *R*_image_(*t*) during the imagery task (Pfurtscheller and Lopes da Silva, 1999):


$$E\,\left(t\right)=\frac{R_{\text{rest}}-R_{\text{image}}\,\left(t\right)}{R_{\text{rest}}}$$

Here, ERD indicates a decrease in μ-wave activity during MI compared to the resting state. Therefore, *E*(*t*) can take values from -∞ to 1 with ERD occurring between 0 and 1.

The ERD feedback, computed using the aforementioned methodology, was visually presented to the participants as a bar graph (Figure 2; Ono et al., 2013). The bar graph changed in real time as the ERD increased or decreased. During the training phase, participants were instructed to imagine the wrist dorsiflexion movement so that the ERD bar displayed on the monitor would increase. The NFB procedure is described in the Supplementary Text and Supplementary Figure 1.

### 2.6. tDCS

**FIGURE 3:**
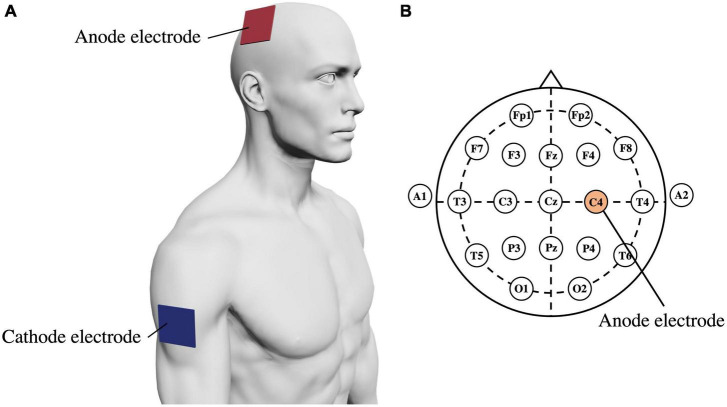
**(A)** Electrode placement for tDCS. The anode electrode is placed over the right primary motor cortex, and the cathode electrode is placed on the right upper arm. **(B)** Electrode placement under the international 10–20 system. The anode electrode is placed on C4.

In the tDCS + NFB group, we applied tDCS for 10 min before the MI task in the training phase. We used a battery-powered stimulator (DC-STIMULATOR PLUS; neuroConn GmbH, Ilmenau, Germany) and a pair of sponge surface electrodes immersed in 0.9% NaCl saline. tDCS increases EEG activity and corticospinal tract excitability related to MI (Ang et al., 2015; Kaneko et al., 2016), thus, ensuring its reliability and validity. The stimulation intensity was set to 2.0 mA and the ramp time to 15 s. The anode electrode (25 cm^2^, stimulation intensity; 2.0 mA) was placed over the right primary motor cortex and the cathode electrode (50 cm^2^, stimulation intensity; −2.0 mA) was placed on the right upper arm (Figure 3; Yamaguchi et al., 2020). Generally, greater stimulation intensity induces greater changes in the electric field (Bikson et al., 2015). However, guidelines for tDCS use indicate that tDCS with an intensity of up to 2.0 mA is safe for humans (Antal et al., 2017). Therefore, we set the stimulation intensity at 2.0 mA, which is safe and maximally effective. In an electric field modeling study, a tDCS of 2.0 mA at M1 produced an electric field change of 0.369 V/m (Evans et al., 2020); a similar electric field change likely occurred in this study. The current density was 0.08 mA/cm^2^ and the total surface charge density was 0.048 C/cm^2^, which is below the threshold for tissue damage (Nitsche et al., 2003).

### 2.7. Evaluation

During both the pre- and post-evaluation phases, as well as the training phase, the mean of the maximum values in each trial was used to evaluate the ERD. In addition, the subjective MI vividness was evaluated using the Visual Analog Scale (VAS) during the pre- and post-evaluation phases. Participants rated the vividness by making a mark on a 100-mm horizontal line, where 0 equaled “not at all” and 100 equaled “very vivid image.” MI vividness correlates with cortical excitability (Moriuchi et al., 2020) and has been used in MI studies involving healthy participants as a simple and subjective MI assessment (Iso et al., 2021; Sawai et al., 2022).

### 2.8. Statistical analysis

First, the normality of all data was examined using the Shapiro–Wilk test. Because a non-normal distribution was detected, unpaired *t*-tests were performed to compare the demographic data of age, height, and weight between groups. Additionally, the Mann–Whitney *U*-test was used to compare ERD values during training between groups. We also compared VAS and ERD values using a two-factor analysis of variance with the factors group (tDCS + NFB group, NFB group) and time (pre, post). The Bonferroni’s method was used for *post-hoc* tests. SPSS ver. 28.0 (IBM Corp., Armonk, NY, USA) was used for all statistical analyses. The statistical significance level was set at *p* < 0.05.

## 3. Results

**FIGURE 4:**
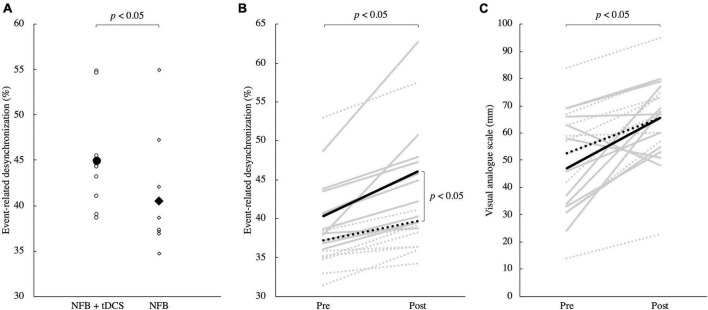
**(A)** ERD in the training phase. Round markers indicate the NFB + tDCS group, and rhombic markers indicate the NFB group. The NFB + tDCS group had significantly higher ERD during MI in the training phase than the NFB group (*p* < 0.05). **(B)** ERD changes between pre- and post-evaluation. The solid line shows the data for the NFB + tDCS group, and the dotted line shows the data for the NFB group. The gray line shows the data for each participant and the black line shows the mean for each group. There was a significant interaction between group and time, with a significant increase in ERD in the NFB + tDCS group compared to that in the NFB group (*p* < 0.05). Furthermore, ERD after training was significantly higher in the NFB + tDCS group than in the NFB group (*p* < 0.05). **(C)** Changes in VAS scores. The solid line shows the data for the NFB + tDCS group, and the dotted line shows the data for the NFB group. VAS increased after training in both groups (*p* < 0.05). MI, motor imagery; NFB, neurofeedback; tDCS, transcranial direct current stimulation; ERD, event-related desynchronization; VAS, visual analog scale.

Demographic data, including age, height, and weight, did not differ significantly between the groups (*p* > 0.05, Supplementary Table 1). ERD during the training phase was significantly higher in the tDCS + NFB group than in the NFB group (*p* < 0.05; Figure 4A). The two-factor analysis of variance for ERD during the evaluation phases revealed a significant interaction between the group and time factors (*F* = 5.16, *p* < 0.05). *Post-hoc* test results showed that ERD in the tDCS + NFB and NFB groups increased significantly before and after training (*p* < 0.05), and that ERD in the post-evaluation phase was significantly higher in the tDCS + NFB group than in the NFB group (*p* < 0.05; Figure 4B). Finally, the two-factor analysis of variance for the VAS revealed a significant main effect of time, with significantly increased values after training (*F* = 16.90, *p* < 0.05; Figure 4C).

## 4. Discussion

This study aimed to investigate the combined effect of tDCS and NFB in MI tasks in healthy young adults. The results showed that the ERD in the NFB + tDCS group during the training phase was significantly higher than that of the NFB group. Additionally, the ERD of the NFB + tDCS group was significantly higher than that of the NFB group in the pre- and post-training phases. Furthermore, the MI vividness was significantly increased before and after training. These results indicate that the combination of NFB and tDCS improves performance in NFB-assisted MI tasks.

The μ-ERD in the training phase of the NFB + tDCS group was significantly higher than that of the NFB group. The μ-ERD in the motor cortex measured in this study indicates the degree of MI (Wei et al., 2013; Kasuga et al., 2015; Xie et al., 2021), suggesting that the addition of tDCS to an NFB-assisted MI task can maximize MI performance. μ-ERD is stronger when MI is successful (Chen et al., 2021), and NFB training provides feedback on the μ-ERD to promote active control, which improves μ-ERD and MI performance. Additionally, tDCS has been shown to increase the probability of neurons firing in response to MI-related input signals (Matsumoto et al., 2010), which in turn increases neuronal depolarization and desynchronization, and enhances μ-ERD. These findings suggest that NFB increases the input signal to the motor cortex *via* MI, and tDCS may further amplify this signal. Therefore, it is likely that the NFB and tDCS mutually contribute to the neural MI activity, resulting in an increased μ-ERD in the training phase of the NFB + tDCS group.

The μ-ERD in the NFB + tDCS group was significantly increased after training compared to that in the NFB group. In the pre- and post-evaluation phases, NFB was not provided in both groups. Therefore, these results suggest that the combination of NFB and tDCS increases the learning efficacy in the post-evaluation phase. NFB has been reported to promote neuroplasticity, resulting in long-term potentiation (LTP) and long-term depression (LTD) of neural networks (Mrachacz-Kersting et al., 2021). Specific neural networks are then modified to become preferential signal input destinations (Yger et al., 2015). Through these actions, NFB facilitates learning. It has also been shown that tDCS, similar to NFB, promotes neuroplasticity and enhances learning (Monte-Silva et al., 2013; Rroji et al., 2015; Barbati et al., 2022). However, tDCS produces a change in electrical polarity that is considerably lower than the potential changes required for depolarization as required for LTP in NFB. Therefore, tDCS promotes its learning effect when activity-dependent LTP is occurring (Fritsch et al., 2010). In this study, μ-ERD after training was significantly improved in the NFB + tDCS group compared to that in the NFB group, which suggests that NFB increased the activity of the neural network related to MI, and tDCS may have reinforced this effect.

On the other hand, the vividness of MI significantly increased in both the NFB and NFB + tDCS groups after training. However, there was no significant difference between the two groups, which indicates that the impact of combining NFB and tDCS on subjective MI evaluation is minimal. Repeated MI improves the vividness of MI (Iso et al., 2021). Nevertheless, studies of MI with simultaneous action observation (Ono et al., 2013) and repetitive peripheral magnetic stimulation (Sawai et al., 2022) reported no difference in the improvement of MI vividness between groups compared to that in MI alone. Similarly, in this study, there was no difference between the NFB and the NFB + tDCS groups. Therefore, the effect of repeated MI on the improvement of MI vividness may be substantial, while the effect of NFB and tDCS on the improvement of MI vividness may be minimal.

There are several limitations to this study: first, the tDCS electrodes used in this study were relatively large (25 cm^2^); thus, we cannot rule out the possibility that other areas adjacent to the motor cortex were affected. Future studies may reveal the effects of tDCS using more targeted stimulation methods. Second, in this study, the task was to imagine a relatively simple dorsiflexion movement of the wrist joint. While μ-ERD was improved in the NFB + tDCS group compared to that in the NFB group, it has been reported that ceiling effects occur in simple tasks, which reduce the effects of tDCS (Furuya et al., 2014). Therefore, the effects of tDCS may be augmented by using more elaborate MI tasks. Third, no group solely received applied tDCS. Therefore, it is unclear whether this result was attributed to the combination of tDCS and NFB or tDCS alone. Fourth, our sample size was small. Therefore, this study is positioned as a feasibility study because of the limited generalizability.

In conclusion, the results of this study indicate that NFB + tDCS increases neural activity not only during, but also after training, and improves MI performance. For immediate MI performance, NFB increased the signal input to the motor cortex, and tDCS increased the firing probability of neurons in response to the signal input. Regarding the learning effect of MI, NFB increases neural activity that produces LTP, and tDCS enhances LTP through subthreshold electrical polarity changes. Therefore, since NFB and tDCS modulate different processes during MI tasks, the concurrent use of these two methods may have augmented MI performance. The present study suggests that this method can be widely applied as a new training technique to improve MI abilities. NFB exerts an inconsistent impact on MI training for the rehabilitation of stroke (Kern et al., 2022) and Parkinson’s disease (Tinaz et al., 2022). Therefore, our results may address this issue. Particularly, the application of our results to patients who have had stroke may help to resolve the lack of improvement in upper limb function (Liu et al., 2012). Further validation in neurological diseases, such as stroke and Parkinson’s disease, as well as in children with cerebral palsy (Behboodi et al., 2022), where the effects of NFB on MI is unclear, will provide more clinical results and advance neurorehabilitation.

## Data availability statement

The raw data supporting the conclusions of this article will be made available by the authors, without undue reservation.

## Ethics statement

The studies involving human participants were reviewed and approved by the Kyoto Tachibana University. The patients/participants provided their written informed consent to participate in this study.

## Author contributions

SS and HN: conceptualization, formal analysis, data curation, writing—original draft preparation, and visualization. SS, SF, RY, and HN: methodology and validation. SS, SM, SF, RY, KS, and HN: investigation and writing—review and editing. SM, KS, and HN: resources. HN: supervision, project administration, and funding acquisition. All authors contributed to manuscript revision, read, and approved the submitted version.
