# Supplementary material for: Effects of neurofeedback training combined with transcranial direct current stimulation on motor imagery: A randomized controlled trial
Source: Front Neurosci. 2023 Mar 2;17:1148336. doi: 10.3389/fnins.2023.1148336 (PMC10017549; doi:10.3389/fnins.2023.1148336)
Supplement: Supplementary file 1 [file Data_Sheet_1.PDF]

Supplementary Material

Effects of Neurofeedback Training Combined with Transcranial Direct Current Stimulation on Motor Imagery: A Randomized Controlled Trial

Shun Sawai [1,2], Shin Murata [1,3], Shoya Fujikawa [3], Ryosuke Yamamoto [4], Keisuke Shima [5], Hideki Nakano [1,3*]

[1] Graduate School of Health Sciences, Kyoto Tachibana University, Kyoto, Japan

[2] Department of Rehabilitation, Kyoto Kuno Hospital, Kyoto, Japan

[3] Department of Physical Therapy, Faculty of Health Sciences, Kyoto Tachibana University, Kyoto, Japan

[4] Department of Rehabilitation, Tesseikai Neurosurgical Hospital, Shijonawate, Japan

[5] Graduate School of Environment and Information Sciences, Yokohama National University, Yokohama, Japan

*** Correspondence:**
Hideki Nakano
nakano-h@tachibana-u.ac.jp

1 Supplementary Table

Supplementary Table S1. Demographic data of each group

	All participants (n=20)	NFB group (n=10)	NFB + tDCS group (n=10)	p-value
Age (years)	20.20 ± 0.70	20.40 ± 0.70	20.00 ± 0.67	0.21
Height (cm)	172.65 ± 6.77	173.70 ± 4.50	171.60 ± 8.60	0.51
Body weight (kg)	63.00 ± 8.52	64.40 ± 5.42	61.60 ± 10.93	0.48

Mean ± SD; NFB, neurofeedback; tDCS, transcranial direct current stimulation

2 Supplementary Text

2.1 EEG Processing

EEG data were analyzed using Microsoft Visual Studio (Microsoft Corp., Redmond, WA, USA). First, a spatial Laplacian filter (Nunez et al., 1994) was used to reduce artifacts, such as eye blink, facial muscle activity, and channel noise. The filtered EEG $y'_l(t)$ for the time t obtained from the electrode l is denoted by the following equation (1) using the pre-filtered EEG $y'_l(t)$, electrodes surrounding the electrode n_l, and the total number of surrounding electrodes N:

$$y'_l(t) = y_l(t) - \frac{1}{N}\sum_{n_l=1}^{N} y_{n_l}(t) \quad (1)$$

After filtering, the EEG was divided into frequencies using filter banks, and the root mean square at each frequency was calculated. The power of each frequency band in the recorded EEG was calculated by the above process, and event-related desynchronization (ERD) was evaluated based on this data. We calculated the ERD $E(t)$ at a time t based on the following equation using the resting μ-wave activity R_{rest} and μ-wave activity $R_{image}(t)$ during the imagery task (Pfurtscheller and Lopes da Silva, 1999):

$$E(t) = \frac{R_{rest} - R_{image}(t)}{R_{rest}} \quad (2)$$

Here, ERD indicates a decrease in the μ-wave activity during MI than that in the resting state. Therefore, $E(t)$ values range from -∞ to 1, with ERD ranging between 0 and 1.

2.2 NFB Procedure

ERD calculated by the above method was visually fed to the participants in real time. A bar graph was projected on the monitor, and the ERD was expressed by an increase or decrease of the graph (Figure S1).

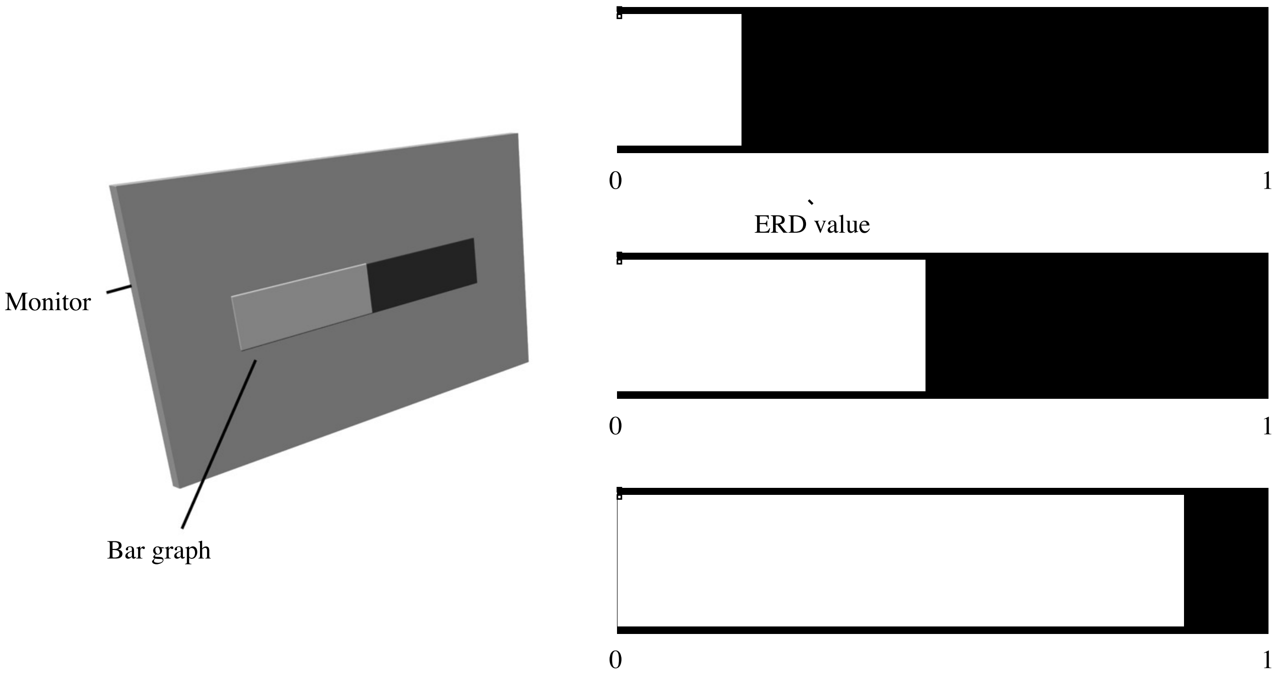

(A) (B)

Supplementary Figure S1. Visual feedback of ERD

(A) A bar graph is projected on the monitor to provide a visual feedback of ERD. (B) The white area on the graph represents ERD, and the graph expands to the right with increasing ERD

ERD: event-related desynchronization
